# TRUST AND BETRAYAL TRAUMA IN OLDER ADULT FINANCIAL EXPLOITATION

**DOI:** 10.1093/geroni/igad104.0882

**Published:** 2023-12-21

**Authors:** LaToya Hall, Peter Lichtenberg, Jennifer Gomez

**Affiliations:** Wayne State University, Detroit, Michigan, United States; Wayne State University, Detroit, Michigan, United States; Boston University, Boston, Massachusetts, United States

## Abstract

Despite a growing body of literature on financial exploitation (FE) and older adults, considerably less research has focused on sub-populations of older adults’ experiences with substantiated financial exploitation, particularly exploitation by trusted others. A 2023 study conducted through Wayne State University’s Successful Aging through Financial Empowerment (SAFE) program used a sample of 95 clients ages 55 and over to examine factors associated with participants’ experiences of substantiated FE perpetrated by trusted others. The SAFE program was created in early 2017 to provide no cost assistance to older adults addressing the financial fall out associated with being financially exploited. The study used betrayal trauma theory (BTT) as a conceptual framework in an attempt to understand how financial vulnerability and mental health are associated with FE committed by trusted others. The study used a cross-sectional design to investigate group differences among 32 (33.7%) older adults who were victims of financial exploitation by trusted others and 63 (66.3%) older adults who were victims of financial exploitation perpetrated by strangers. The group of older adults who were victims of FE by trusted others had lower functional ability scores, higher stress and financial exploitation vulnerability scores and lost more money on average than those victimized by strangers. These results suggest older adults who have been victimized by trusted others are more vulnerable and need more assistance with functional tasks than those victimized by strangers. The present study also supports the notion that BTT provides a valuable framework for understanding vulnerability to various types of FE.

